# β3-Adrenoreceptors as ROS Balancer in Hematopoietic Stem Cell Transplantation

**DOI:** 10.3390/ijms22062835

**Published:** 2021-03-11

**Authors:** Amada Pasha, Maura Calvani, Claudio Favre

**Affiliations:** 1 Division of Pediatric Oncology/Hematology, Meyer University Children’s Hospital, 50139 Florence, Italy; amada.pasha@unifi.it (A.P.); claudio.favre@meyer.it (C.F.); 2 Department of Health Sciences, University of Florence, 50139 Florence, Italy

**Keywords:** β3-adrenoreceptor, antioxidant activity, hematopoietic stem cells

## Abstract

In the last decades, the therapeutic potential of hematopoietic stem cell transplantation (HSCT) has acquired a primary role in the management of a broad spectrum of diseases including cancer, hematologic conditions, immune system dysregulations, and inborn errors of metabolism. The different types of HSCT, autologous and allogeneic, include risks of severe complications including acute and chronic graft-versus-host disease (GvHD) complications, hepatic veno-occlusive disease, lung injury, and infections. Despite being a dangerous procedure, it improved patient survival. Hence, its use was extended to treat autoimmune diseases, metabolic disorders, malignant infantile disorders, and hereditary skeletal dysplasia. HSCT is performed to restore or treat various congenital conditions in which immunologic functions are compromised, for instance, by chemo- and radiotherapy, and involves the administration of hematopoietic stem cells (HSCs) in patients with depleted or dysfunctional bone marrow (BM). Since HSCs biology is tightly regulated by oxidative stress (OS), the control of reactive oxygen species (ROS) levels is important to maintain their self-renewal capacity. In quiescent HSCs, low ROS levels are essential for stemness maintenance; however, physiological ROS levels promote HSC proliferation and differentiation. High ROS levels are mainly involved in short-term repopulation, whereas low ROS levels are associated with long-term repopulating ability. In this review, we aim summarize the current state of knowledge about the role of β3-adrenoreceptors (β3-ARs) in regulating HSCs redox homeostasis. β3-ARs play a major role in regulating stromal cell differentiation, and the antagonist SR59230A promotes differentiation of different progenitor cells in hematopoietic tumors, suggesting that β3-ARs agonism and antagonism could be exploited for clinical benefit.

## 1. Introduction

### Hematopoietic Stem Cell Transplantation

**Figure 1:**
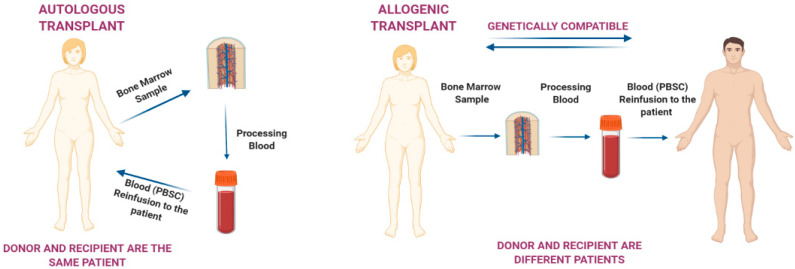
Difference between autologous and allogenic transplants.

Hematopoietic stem cells (HSCs) and progenitor cells (HSPCs) are a small population of undifferentiated cells localized in the bone marrow (BM). They can sustain self-renewal by giving rise to mature cells; moreover, under stress conditions, they migrate from the BM to the peripheral blood (PB) [1]. This process is enhanced as a part of the host’s defense and repair mechanisms. Multipotent HSCs, which are used for transplantation, are usually harvested from the PB, BM, and umbilical cord blood [2]. HSCs infused to the blood stream during the process of hematopoietic stem cell transplantation (HSCT) can return to the BM and restore the HSC pool, providing a lifelong source of new blood and immune cells [3]. In the last decades, HSCT has become an attractive therapeutic approach for different diseases such as cancer, hematologic conditions, immune system dysregulations, and inborn errors of the metabolism [4]. HSCs can be used in autologous, allogeneic, and syngeneic transplantations, when the stem cells are derived from the same patient, a donor, or an identical twin, respectively (Figure 1) [5]. Patients with blood or BM cancer, such as leukemia or multiple myeloma, are treated with HSCT [6]. Despite being a promising therapeutic option, HSCT can also lead to severe adverse effects. Among them, acute and chronic graft-versus-host diseases (GvHD) are the principal complications of allogeneic HSCT, but hepatic veno-occlusive disease, lung injury, and infections are of clinical interest as well. Infections can be extremely threatening in the early stages after transplantation considering the risk of reactivation of well- and less-known pathogens [7]. Despite its associated risks, HSCT allowed to improve survival rates. However, its use was not only restricted to different forms of cancer such as neuroblastoma, lymphoma, Ewing’s Sarcoma, Hodgkin’s disease, but it has also been extended to chronic granulomatous disease [8], autoimmune diseases, mucopolysaccharidosis (metabolic disorder) [9], malignant infantile disorders, and hereditary skeletal dysplasia [10]. Nowadays, the prevalent source of stem cells for HSCT are peripheral blood cells (PBCs) [11] which are collected from the blood through the process of apheresis. The major curative therapy used to treat several disorders such as autoimmune diseases, metabolic disorders, hemoglobinopathies, immunodeficiencies and many others for over 30 years is HSCT [12,13]. HSCT has become the most appropriate therapy for a wide variety of hematological and non-hematological diseases including leukemia [4], lymphomas, anemias, and immunological and genetic disorders [14]. HSCT is performed to restore or to treat various congenital conditions in which immunologic and hematologic functions are absent or impaired after a chemotherapy and a radiation therapy [15]. Further, it implies the administration of healthy HSCs in patients with dysfunctional or depleted BM. The goal of this therapeutic approach is to allow long-term improvement of the disease, and to supply an adequate percentage of marrow engraftment [13]. The type of the transplant used depends on the disease, and may include autologous, allogeneic, marrow stem cells, or cord transplantation, which can help to enhance BM function to achieve an anti-tumor immunity to produce functional cells that can restore the dysfunctional ones in immune-deficiency syndromes [14]. The time of transplantation depends on the clinical severity of the disease. It is important to determine the clinical parameters that define the severity of the disease to confirm whether HSCT is indicated. 

During the 1980s, HSCT increased worldwide [16]: over the past 36 years, more than 1300 patients with various forms of genetical immunodeficiencies have undergone HSCT to repair their underlying immune defects [17]. The results of HSCT improved significantly over the last two decades, possibly also due to early diagnosis before the development of infections and through the availability of better antimicrobials for infections treatment [18]. HSCT has been extended to adult populations due to the early success in pediatric populations. For this reason, nowadays, the majority of HSCTs are performed in adults [19]. In the past two decades, due to faster engraftment and practicability, peripheral blood stem cell transplantation (PBSCT) replaced BM as a stem cell fount [20]. There appeared to be two main problems associated with BMT: rejection and GvHD. In a study of 2005, PBSCT was associated with a reduced relapse percentage in patients with late-stage diseases (hematological malignancies), a rapid neutrophil, and platelet engraftment. However, PBSCT was also correlated with a higher risk of developing GvHD than BMT [21]. Half of the transplant doctors prefer to perform allogeneic PBSCT than BMT [22]. The physician has to consider both benefits and drawbacks correlated with each of the treatment alternatives when choosing between two treatment interventions. When deciding which stem cell source to use, both doctors and patients should consider the higher risk of disease recurrence with BMT against the long-term consequences of chronic GvHD [23]. 

## 2. Reactive Oxygen Species

ROS include a heterogeneous group of small molecules and free radicals, which contain oxygen atoms with unpaired electrons in their outer valence shell [24]. They are produced by internal oxygen metabolites that have polar molecules such as superoxide anion (O_2_⋅-), hydroxyl radical (⋅OH), hydroperoxyl radical (HO_2_), peroxyl radical (RO_2_⋅), alkoxyl radical (RO-), and non-polar molecules such as hydrogen peroxide (H_2_O_2_). ROS are produced by numerous enzymatic systems, including the NADPH (nicotinamide adenine dinucleotide phosphate) oxidases and the mitochondrial electron transport chain. [25]. If a condition of homeostasis between oxidative species and antioxidants is not maintained, oxidative stress (OS) can arise, resulting in an increase in oxidant processes due to a lack of antioxidant defense. These phenomena can induce tissue injuries and activate cell pathways involved in cancer progression [26]. The OS reaction is induced by an increase in ROS levels and/or damage of antioxidant systems. High ROS levels can cause cellular DNA damage and cell-cycle arrest [27]. These active radicals can damage important cellular components such as DNA, alter gene expression, induce changes in the activity of critical metabolites, and determine the survival or death of cells [28]. Two common hallmarks of tumors strongly implicated in malignant progression and resistance to treatments are the altered redox balance and deregulated redox signaling [29]. ROS are involved in innate immunity and inflammatory signaling through pathogen elimination, although their exact functions within the complex metabolic network are unclear [30]. Cardiovascular diseases, cancer, atherosclerosis, diabetes, and neurological and endocrinological disorders have been associated with OS upregulation caused by excessive production of ROS or by decreased scavenging contribution [31,32]. To maintain redox homeostasis, cells have developed various sophisticated mechanisms to neutralize the deleterious effects of ROS. The antioxidant ability of the cells appears to be a strong developmental feature to counteract the damage caused by OS [33]. In case of a small increase in ROS levels, the antioxidant response can balance the ROS levels and restore the equilibrium between ROS production and scavenging ability. Tumor cells can regulate multiple enzymes and use their metabolic pathways (antioxidant enzymes in conjunction with non-enzymatic antioxidants such as glutathione, thioredoxin, and vitamins A, C, and E) to provide an adequate supply of antioxidant systems. Superoxide dismutase, catalase, and glutathione peroxidase are the first defense in the endogenous neutralization of ROS. The peroxiredoxin, thioredoxin, and glutathione/glutaredoxin systems are also mediators of redox signaling and constitute another important cell defense mechanism [34,35,36].

The glutathione/glutaredoxin systems and peroxiredoxin are two mechanisms whose expression is activated by nuclear factor erythroid 2-related factor 2 (Nrf2). There are two types of antioxidants: direct antioxidants with redox activity and short half-lives, and indirect antioxidants, whose physiological effects last longer [37]. They act through an increase in the cellular antioxidant capacity by enhancing the expression of specific genes, such as *NFE2L2* encoding for Nrf2, a master regulator of the antioxidant [38].

### HSCT and ROS

Self-renewal and multi-potent differentiation are two of the basic properties of HSCs [39]. The function of HSCs is regulated by both intrinsic and extrinsic factors: the intrinsic factors arise from signaling pathways in HSCs, whereas the extrinsic factors arise from multiple factors such as the BM niche, the microenvironment where HSCs reside [40]. Most of the HSCs are quiescent and nonmotile within the hypoxic niche [1]. The BM niche includes different types of components such as cells, extracellular matrix, cytokines, blood vessels, and adhesion molecules. Distinct blood vessels with different permeability properties regulate the maintenance of the BM stem cells. The high permeability of blood vessels promotes HSCs activation and an increase in bone marrow hematopoietic stem cell ROS levels, stimulating cell migration and differentiation [41].

The ROS are another metabolic niche factor that has received attention [42]. In normal conditions, increased glycolytic activity reduces mitochondrial oxidation and decreases the levels and ROS production [43]. Since HSCs are susceptible to OS, it is important to maintain their self-renewal capacity [44]. In the normal hematopoiesis process, ROS regulate migration and myeloid differentiation of HSCs [45]. Primitive HSCs that reside in a hypoxic BM environment generate energy by maintaining a high rate of glycolysis via anaerobic glycolysis [42]. This hypoxic BM microenvironment protects HSCs from the OS that would otherwise inhibit their self-renewal and result in BM failure [46]. The crosstalk between HSCs and the BM microenvironment is important for normal hematopoiesis. 

Quiescent HSCs maintain low levels of ROS to maintain their stemness features. However, to promote HSC proliferation and differentiation, it is important to maintain physiological ROS levels [47]. Cells with high ROS levels are mainly involved in short-term repopulation, whereas cells with low ROS levels have better long-term repopulating capacity [48]. In adults, and during embryonic development, moderate levels of ROS are essential for hematopoietic homeostasis [49], whereas high ROS levels may induce significant defects and eventual depletion of HSCs [50]. It is known that high ROS levels constitute a risk factor for HSCT in both experimental and clinical settings. The ROS may be involved in the initiation and progression of myelodysplastic syndromes and acute myeloid leukemia [51]. ROS play an important role in hematopoiesis through the effects mediated by the production and secretion of the cytokine CXCL12 of stromal cells; moreover, they can stimulate the expression of CXCL12 on BM stromal cells, which promotes HSC migration and retention in the hematopoietic niche [52]. Physiological ROS levels are associated with an increased expression of CXCR4 on HSCs, providing conditions for their migration to BM stromal cells.

Bai et al. investigated if the ROS levels correlate with neutrophil and platelet engraftment after autologous HSCT; they found that high ROS levels did not correlate with platelet engraftment [45].

In experiments with mouse models, elevated ROS levels showed the highest negative impact on HSCT. After total body irradiation (before transplantation), a high production of ROS was observed that can induce a sort of bystander effect on transplanted HSCs [53]. In quiescent, proliferating, or differentiating stem cells, different amounts of intracellular ROS are exhibited due to their different metabolisms. ROS have a beneficial effect on stem cells if maintained in a strict concentration range. If is exceeds this range, it would lead to senescence of the stem cell and the loss of its functionality [1]. ROS can be involved in the initiation and progression of hematopoietic malignancies, such as myelodysplastic syndrome and acute myeloid leukemia, by inducing unspecific oxidative damage of DNA, lipids, and proteins or by hyperactivation of ROS signaling pathways [54]. Redox regulation of mammalian HSCs is under intense examination since they are more vulnerable to ROS than the progenitors, and tend to lose their stemness and die after ROS exposure [42]. 

The OS is a notorious inducer of autophagy. It is an important component of the transplantation process and seems to play an important function in oxidative signals during transplantations. OS and autophagy are both affected by the stress responses triggered in each step (donor, preservation, and recipient) of the transplantation process [55].

## 3. β3-Adrenoreceptor

The β3-AR, also known as ADRB3 as the human gene encoding it, is the last identified member of the β-ARs family. The β-ARs family also includes β1- and β2-Ars, which are widely found in different types of tumors, such as brain, lung, liver, kidney, adrenal gland, breast, ovary, prostate, or lymphoid tissues [56]. The ADRB3 gene is localized on chromosome eight in humans, and it shares a 51% and 46% identity with β1- and β2-AR amino-acid sequences, respectively; the sequence homology is mostly limited to the transmembrane domains and membrane-proximal regions of the intracellular loops [57]. The third subtype, β3-AR, was cloned for the first time in 1989 [58], and it differs from β1- and β2-ARs in both molecular structure and pharmacological profile, suggesting a different intracellular signaling pathway.

The isotype β3-AR was initially found abundantly expressed in white and brown adipose rodent tissue, where it mediates lipolysis and thermogenesis [59,60]. Subsequently, it was described as playing a crucial role in the pathophysiology of the cardiovascular system [61] and urinary tract [62]. β3-AR expression has been recorded both at mRNA and protein levels and its presence has been reported across different tumors whose activation involves a variety of cellular pathways, including vascular tumors, human leukemia cells, colon, and breast cancer [63,64,65,66]. In humans, β3-ARs have a more restricted expression pattern compared with β1- or β2-ARs [67] due to a unique and species-dependent ligand recognition profile [68]. Recently, preclinical evidence supports a possible role of β3-ARs in melanoma. In a recent work, Calvani et al. demonstrated that there might be an additional dimension with upregulation of β3-ARs in subpopulations of immune cells [69].

Adrenaline and noradrenaline act as β-adrenergic receptor ligands and drive the development of tumor growth and metastasis through the modulation of cell proliferation and apoptosis [70]. Quantitative analysis of the human transcriptome showed that β3-AR expression is far more restricted than previously hypothesized [71]. β-ARs are proposed as a potential target for a therapeutic approach to cancer, since several studies suggested that stress-related catecholamine release accelerates cancer progression [72]. β3-AR expression is associated with cancer progression, angiogenesis, and tumor-stromal cell reactivity [65]. Many types of cancer proved to have an overexpression of β-ARs and their pharmacological inhibition with β-blockers as anticancer agents showed clinical efficacy. This suggests that β-blockers can contribute to the improvement in the survival and decrease in tumor proliferation and progression in many cancer types [66,73]. The screening for new compounds is progressing at a fast rate since β3-ARs are gaining more and more importance as therapeutic targets [74].

In the last few years, the first selective β3-ARs agonist, mirabegron, was developed and approved for its effect on reducing the bladder and detrusor muscle tone in vitro in overactive bladder syndrome [75]. β3-AR agonists are divided into two classes depending on the time of their discovery [76]: the first-generation compounds (such as BRL37344 and CL316243) were developed in the 1990s; the second-generation compounds were developed later. The most-used β3-AR antagonists are SR59230A and L748337.

This receptor is an interesting target for novel therapeutic approaches and targeting β3-AR has been proposed for the treatment of several conditions, with some drugs already undergoing Phase II and III clinical trials due to advances over the last two decades [77].

### 3.1. β3-ARs and ROS

Recently, it was shown that β3-AR exerts a dual role in antioxidant cell response: it directly inhibits NADPH oxidase activity and induces the expression of catalase, an enzyme that plays a crucial role as an endogenous antioxidant [78]. Noradrenaline induces the catalytic subunit of glutamate–cysteine ligase protein, which increases the intracellular glutathione (GSH) levels through the stimulation of β3-AR in U-251 MG cells and mouse astrocytes in primary culture [79]. ROS induce GSH synthesis, which is essential for the protection of neurons from oxidative damage. Calvani et al. showed that β3-AR expressed in melanoma cells drives the activity of uncoupling protein-2 (UCP2), piloting the ROS content in the mitochondria [80]. This study demonstrated the β3-AR antioxidant activity and its role as a mediator for the increase in endogenous antioxidants activity of the cells. β3-AR could work as an ROS sensor controlling the redox state of the cells, driving cells to life or death through mitochondria bioenergetics function [81] by identifying the β3-AR receptor as the main regulator of the cellular response to OS. Pasha et al. observed that the nutraceutical antioxidant apigenin induced partial cell death in Ewing Sarcoma (ES) cell lines by inducing the activation of the apoptotic pathway without increasing the mitochondrial ROS production (mtROS), which instead is evident in the administration of the β3-AR antagonist, SR59230A. Apigenin inhibits the expression of antioxidant proteins (superoxide dismutase 2, catalase, sirtuin-1, thioredoxin, thioredoxin-interacting protein, glutathione S-transferase Mu4, and Nrf2), but increases UCP2 and GSH levels. The antioxidant activity of β3-ARs could be mediated by UCP2 protein expression, which could control the redox homeostasis in ES cells [82].

In a recent study, Calvani et al. demonstrated that cells treated with curcumin, 8-gingerol, genistein, and retinoic acid showed reduced viability compared with cells treated with capsaicin, ascorbic acid, formononetin, and flavon, which did not affect the cell viability [81]. Moreover, low levels of intracellular mtROS were observed in cells treated with prosurvival antioxidants, whereas an increase in mtROS levels were evident in cells treated with antioxidants that can reduce cell viability. β3-ARs expression was u-regulated when cell viability was not affected by the treatment, and the treatment that reduced cell viability strongly downregulated the β3-ARs levels. These results identified the β3-ARs as one of the main regulators of the cellular response to OS under treatment with different micronutrients. β3-ARs function as ROS sensors in ES cells by inducing, or not inducing, antioxidant response and cell death. As the antagonism of β3-ARs leads to massive cell death, inhibiting β3-ARs in these cells could dramatically increase the ROS levels above the toxic threshold, leading to cell death.

In adipose tissue, β3-AR stimulation displays the same effects as stimulation of the transcription factor PPARγ, which has commonly been described to exert antioxidant properties [83,84]. Moreover, it was demonstrated that β3-AR can induce the over-expression of PPARγ [85]. The antioxidant effects of β3-AR in human macrophages led to potent anti-inflammatory effects [78]. 

### 3.2. β3-ARs and HSCs

Every day, billions of blood cells are produced during the process of hematopoiesis, which occurs in the BM by HSC proliferation and differentiation. HSC niches are regulated by sympathetic nerves, a variety of mature hematopoietic cells (such as macrophages, neutrophils, and megakaryocytes), and non-myelinating Schwann cells [86]. Adrenergic receptors are expressed by many cellular components of the niche, and the BM cells can produce and release catecholamines [87]. The first evidence that BM function may be influenced by an adrenergic system was found after the 1990s [88]. In 1997, Marino et al. showed that the catecholamines, via α1-AR, may influence hematopoiesis and that BM contains high amounts of catecholamines due to the secretion of sympathetic post-ganglionic fibers and endogenous of the BM cells [89]. Moreover, the α-adrenergic antagonists may highly increase myelopoiesis and platelets production while decreasing lymphopoiesis after HSCT. In the BM, β-AR stimulation may result in a variety of effects such as after burn injury if there is an increase in myelopoiesis in situations of increased sympathetic nervous system activity [90]. Under stable conditions, a small amount of HSPC leaves the BM and penetrates the tissues, and returns via the blood or lymphatic system to the BM or peripheral niches [91]. The mobilization is the release of HSPC from the BM into the peripheral blood, and it may be a danger-sensing response mechanism triggered by hypoxia or tissue injury [92]. Katayama et al. showed the involvement of an adrenergic mechanism in HSP mobilization [93]. 

Studies on circadian rhythms provided the first evidence of sympathetic regulation of hematopoiesis. Mendez-Ferrer et al. observed that continuous exposure to light or jet lag altered the number of HSCs in the mouse BM, indicating that photic cues could influence the trafficking of HSCs [94]. 

**Figure 2:**
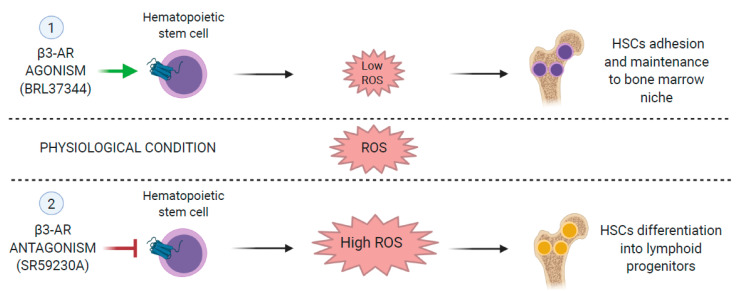
Effects of β3-adrenoreceptors (Ars) agonists/antagonists on ROS levels and hematopoietic stem cells (HSCs) differentiation.

Calvani et al. demonstrated that SR59230A, a β3-AR antagonist, promotes hematopoietic differentiation by increasing the ratios of lymphoid/HSCs and myeloid progenitor cells/HSCs by increasing the number of Ter119, natural killer (NK) precursor cells, and granulocyte precursors [95]. Additionally, pharmacological antagonism of β3-AR induces mesenchymal stem cell differentiation into adipocytes. Moreover, β3-ARs induce the recruitment of stromal cells in the tumor microenvironment [65] as β3-AR was shown to regulate stromal cell differentiation. Antagonism with SR59230A can promote the differentiation of progenitor cells in hematopoietic tumors (Figure 2) [95]. The β-adrenergic system was identified as one of the principal players in the regulation of the immune system. β_2_-adrenergic stimulation inhibits lymphocyte responses, natural killer (NK) cytotoxicity, and dendritic cell functions [96]. Calvani et al. evaluated the potential role of β_3_-ARs in the regulation of melanoma immuno-tolerance by pharmacological approach [69]. They showed that both β_2-_ and β_3_-ARs were expressed in mouse PBMCs, but under the hypoxic condition, only β_3_-ARs showed a reversible upregulation. Moreover, compared with circulating cells, β_3_-ARs were remarkably up-regulated in NK, Treg, and myeloid-derived suppressor cell (MDSC) infiltrating the tumor. The antagonism of β_3_-adrenoceptor could reduce melanoma growth in vivo by attenuating Treg and myeloid-derived suppressor cell (MDSC) sub-populations in the tumor microenvironment and by increasing the number of NK and CD8 cells [69]. Moreover, β_3_-ARs blockade could induce a shift in macrophage and neutrophil phenotypes from both M2 to M1 and N2 to N1. Since hypoxia is considered one of the most important regulators of cancer immune-tolerance, the hypothesis that β_3_-ARs might be involved in the acquisition of an immune-tolerant phenotype under hypoxic conditions is supported [97]. β_2_- and β_3_-ARs are expressed in T lymphocytes; among them, β_3_-ARs are upregulated in response to stress [98]. The β_3-_AR antagonist, SR59230A, can increase NK and CD8 cells with a strong reduction in Treg cells and MDSC within the tumor mass, counteracting melanoma growth in vivo [69]. The authors showed the involvement of β_3-_ARs in immune tolerance, reinforcing the hypothesis that it could be a target for a melanoma growth control therapy.

The adrenergic modulation of hematopoiesis has considerable potential for pharmacological therapeutic approaches in hematopoietic disorders and HSCT. However, due to the complexity of the system, further studies are needed [86].

## 4. Conclusions

HSCT has emerged as a therapeutic approach that generates healthy and differentiated cells, and repairs deteriorated or damaged tissues and organs. ROS may influence many biological processes, but the knowledge of which ROS are implicated in any given physiological setting is limited. Studying ROS metabolism, how it can be manipulated to generate stem cells, and its influence on the stem cell fate could be challenging. An increased ROS level correlates with mammalian blood stem cell differentiation and increased production of their immediate progenitors, where ROS mediates cell cycle progression. Blood stem cell activity reduction occurs within regions of the BM that have increased levels of ROS [95]. Low ROS levels are essential to maintain the stemness of quiescent HSCs, but a physiological level of ROS is needed to promote HSC proliferation and differentiation. β3-ARs antagonist SR59230A induces hematopoietic differentiation of lymphoid/HSCs, myeloid progenitor cells/HSCs [95], and mesenchymal stem cell differentiation into adipocytes, reducing the potential renewal of the stem compartment of these cells [65]. In this review, we aimed to suggest that the β3-AR agonist could be tested in the first phase of transplantation to maintain low to moderate levels of ROS, and to preserve stemness and long-term differentiation. When HSCs start to differentiate, ROS must be kept at moderate/high levels to stimulate hematopoietic differentiation, as previously demonstrated in melanoma [96,99]. However, as the molecular biomarkers have not yet been identified, it is hard to state the necessary dose and timing of agonists/antagonists’ administration. An accurate determination of the dose and the timing administration is a problem yet to be solved, so more studies are needed. The adrenergic modulation of hematopoiesis holds considerable potential for pharmacological therapeutic approaches in hematopoietic disorders and HSCT [86]. Since β3-ARs represent a promising therapeutic target of systemic disorders, studying the pharmacological effects of β3-ARs’ agonists and antagonists could represent an exciting opportunity for future explorations.

## Abbreviations

BM	Bone marrow
β3-AR	β3-adrenoreceptor
GSH	Glutathione
GvHD	Graft-versus-host disease
HSCs	Hematopoietic stem cells
HSCT	Hematopoietic stem cell transplantation
HSPCs	Hematopoietic progenitor stem cells
MDSC	Myeloid-derived suppressor cell
mtROS	Mitochondrial ROS
NADPH	Nicotinamide adenine dinucleotide phosphate
NK	Natural killer
OS	Oxidative stress
PB	Peripheral blood
PBCs	Peripheral blood cells
PBSCT	Peripheral blood stem cells transplantation
ROS	Reactive oxygen species
UCP2	Uncoupling protein-2
